# Characterization and Design of Photovoltaic Solar Cells That Absorb Ultraviolet, Visible and Infrared Light

**DOI:** 10.3390/nano11010078

**Published:** 2021-01-01

**Authors:** Sara Bernardes, Ricardo A. Marques Lameirinhas, João Paulo N. Torres, Carlos A. F. Fernandes

**Affiliations:** 1 Department of Electrical and Computer Engineering, Instituto Superior Técnico, 1049-001 Lisbon, Portugal; sarabernardes@tecnico.ulisboa.pt (S.B.); joaotorres@tecnico.ulisboa.pt (J.P.N.T.); ffernandes@tecnico.ulisboa.pt (C.A.F.F.); 2 Instituto de Telecomunicações, 1049-001 Lisbon, Portugal; 3 Academia Militar, Av. Conde Castro Guimarães, 2720-113 Amadora, Portugal

**Keywords:** concentrator systems, GaInP/GaInAs/Ge, multi-junction, photovoltaics, solar cells, space, triple-junction

## Abstract

The world is witnessing a tide of change in the photovoltaic industry like never before; we are far from the solar cells of ten years ago that only had 15–18% efficiency. More and more, multi-junction technologies seem to be the future for photovoltaics, with these technologies already hitting the mark of 30% under 1-sun. This work focuses especially on a state-of-the-art triple-junction solar cell, the GaInP/GaInAs/Ge lattice-matched, that is currently being used in most satellites and concentrator photovoltaic systems. The three subcells are first analyzed individually and then the whole cell is put together and simulated. The typical figures-of-merit are extracted; all the I−V curves obtained are presented, along with the external quantum efficiencies. A study on how temperature affects the cell was done, given its relevance when talking about space applications. An overall optimization of the cell is also elaborated; the cell’s thickness and doping are changed so that maximum efficiency can be reached. For a better understanding of how varying both these properties affect efficiency, graphic 3D plots were computed based on the obtained results. Considering this optimization, an improvement of 0.2343% on the cell’s efficiency is obtained.

## 1. Introduction

The constant search for new energetic solutions to face the ever-demanding world’s energy consumption has been one of the main focus amongst researchers in the twenty-first century. At the time this article is being written, a good and affordable alternative seems to be found in the use of renewable energies [1,2,3,4,5,6,7,8,9,10,11,12,13,14,15,16,17,18,19]. Even though the world is not yet prepared to switch completely to renewable sources, the installed capacity of these sources is increasing day by day, with the global renewable generation power already surpassing 2300 gigawatts. In 2018, 20% of this total generation capacity came from solar power, that continued to dominate in terms of new power installed, representing an increase of 24% [1].

This global solar expansion mainly derives from the capability of the photovoltaic (PV) industry to face the challenges that have been proposed until now.

In the years to come, PV has the capacity of becoming one of the major energy sources in the world—as the price of fossil fuels continuously rises [6,7,8,9,10,11,12,13,14,15,16,17,18,19], the cost of solar PV has been substantially decreasing over the last two decades, with its LCOE (Levelized Cost Of Energy) being estimated to be within the range of 0.03 to 0.10 $/kWh by 2020–2022 [2,3,20]. This prophetizes a solid future for the PV industry, especially if it is supported by the decrease in battery prices.

All of this motivates the industry to come up with new and improved solutions; one of those improvements in recent decades is the use of III-V multi-junction solar cells. These photovoltaic devices employ III-V semiconductors (made of elements in groups III and V of the Periodic Table), typically in a stacked distribution [4,5,21,22]. These cells have been demonstrating solid results in terms of efficiency, since the first III-V GaInP/GaAs tandem cell was demonstrated by Olson and Kurtz at NREL in 1996, with a record efficiency of more than 30% [21]. Today, III-V cells already hit the mark of 45.7% in concentrator photovoltaics (NREL, 4-junction GaInP/GaAs/GaInAs/GaInAs, 234 suns) [22], demonstrating extraordinary advances in choosing optimal bandgap distributions.

This work will focus on a specific III-V cell, the GaInP/GaInAs/Ge lattice-matched cell, the state-of-the-art cell for both concentrator photovoltaics and space applications. The main objective is then to build a simulation model that allows for a characterization of the subcells that form the whole cell, extracting I−V curves and external quantum efficiencies, along with the most relevant figures-of-merit, such as fill-factors and efficiencies. A general optimization of the cell will also be attempted; this will be done by altering the thickness and doping of some layers.

It is used a Finite Element Tool, for being capable of simulating a 2D and 3D solar devices by providing a large set of physical models (drift-diffusion, general optoelectronic interactions with ray tracing, Fermi-Dirac statistics, etc.) for semiconductor device simulation.

## 2. Fields of Application of III-V Solar Cells

**Figure 1:**
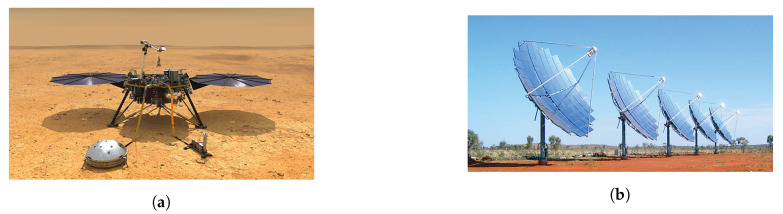
Some examples of solar cells use in space and terrestrial applications: (**a**) NASA’s InSight Lander robot, powered by solar energy, and already owns the off-world record of power generation. (**b**) A HCPV parabolic system that uses high-efficiency multi-junction modules by Solartron Energy Systems.

The III-V MJ (multi-junction) solar cells are utilized in the most varied fields of application, the most important two being space applications and concentrator photovoltaic (CPV) systems, as illustrated respectively in Figure 1a,b. These two fields represent very different operating conditions for solar cells, and thus different design approaches for each field must be considered. Record efficiencies of 35.8% (AM0 spectrum) [23] and 46% (AM1.5d spectrum, 508 suns) [22] were already demonstrated for space and CPV applications, respectively.

### 2.1. Space Applications

Regarding space applications, III-V cells have become the go-to technology, not only because of their high-efficiency results but also because of their high tolerance to radiation exposure. After being irradiated with high radiation doses, these cells showed an EOL (end-of-life) efficiency that was higher than a BOL (beginning-of-life) efficiency of a standard Si solar cell. Of course, this represented a major change for the spacecraft industry, since a good EOL efficiency is intrinsically connected to the weight and cost of the overall system, paramount factors when discussing the launch of a spacecraft, in which the cost is determined by €/kg, as opposed to €/W_p_ in terrestrial applications.

Therefore, these cells, given their high EOL efficiencies, good radiation tolerance, and high power-to-mass ratios (W/kg), meet the requirements of the majority of the NASA OSS (National Aeronautics and Space Administration Operational Support Services) missions, that call for high specific power values, making them the state-of-the-art cells for the majority of satellites and space vehicles.

Another important aspect concerning missions in space is the temperature at which PV modules must operate in certain harsher environments. Space PV arrays must be prepared to endure both high and low temperatures, depending on the mission’s orbit. This leads to the necessity of studying the cell’s temperature coefficient (dη/dT) to have a measure on how the performance of the cell will vary with temperature. When under the AM0 spectrum, the normalized temperature coefficient of a Si solar cell is in the range of $-3\times 10^{-3}$ /${}^{{}^{\circ}}C$ to $-5\times 10^{-3}$ /${}^{{}^{\circ}}C$, while for tandem GaAs/Ge cells the temperature coefficient is approximately $-2\times 10^{-3}$ /${}^{{}^{\circ}}C$ [24].

This notorious difference in temperature coefficients is explained by the variance of bandgap in both cells; solar cells that have in their composition materials with higher bandgap values show lower efficiency losses with temperature [25]. This means that there will be an ideal bandgap for each operating temperature.

### 2.2. Terrestrial Concentrator Systems

On Earth, the task of implementing III-V plate modules would represent a heavy cost of production, with the cost of a typical III-V high-efficiency cell being around $10\;\$/{\mathrm{cm}}^{2}$ [26]. To counter this problem, solar PV companies developed concentrator photovoltaic systems (CPV), in which sunlight is concentrated with the use of mirror lenses. Usual concentration ratios for III-V cells may go from 500× to 2000×, the latter being commonly called high concentration PV (HCPV).

The increase in irradiance will directly affect the short-circuit current of the cell, increasing it. Resorting to Equation (1), it is easy to see that incrementing $I_{SC}$ affects the open-circuit voltage of the cell, which increases logarithmically by several KT/q factors. This boost in the $V_{OC}$ will be more evident for a multi-junction cell, in which every subcell will contribute for the increase of $V_{OC}$ with concentration, and thus rising the fill-factor of the overall cell [6,7,8,9,10,11,12,13,14,15,16,17,18,19].

(1) $$\begin{array}{c} V_{OC}=nV_{T}\ln\frac{I_{SC}}{I_{0}}+1 \end{array}$$

For this reason, it would be fair to think the higher the concentration ratio, the higher the efficiency of the cell. Alas, in reality, no device is ideal, including solar cells; there are always losses that need to be considered, such as series and shunt resistances that must be taken into consideration. The concentration increase will have a dominant impact on the overall efficiency, diminishing the FF (Fill Factor), and changing the I−V characteristic. The greater the concentration ratio, the higher the impact will be on the cell; e.g., for the TJ (triple-junction) GaInP/GaInAs/Ge, when incrementing the series resistance from $R_{s}=0$ to $R_{s}=0.1\;\Omega$, the FF is reduced from 90% (1 sun) to 87% at 83 suns, and to 71% at 500 suns [27].

Analyzing this data, it was then evident that some changes in series and shunt resistances had to be made in such a way that cells could operate under high concentration levels so that losses could be, to an extent, negligible. Every concentrator cell has a concentration limit for which the efficiency will start to drop, and several studies are being conducted in this matter. In the work of Steiner et al. [28], three tests were made using the single junction GaAs solar cell to prove the reduction in the FF and efficiency: three optimized grids for concentrations of C=100, C=450, and C=1000 were tested, and the cell showed a maximum efficiency of 29.09% for a concentration of 450 suns.

## 3. III-V Solar Cell Design

For a better understanding of the fundamentals behind a III-V solar cell it is necessary to perceive where they differ from the simple junction cell. It has already been stated that III-V multi-junction cells are top performers in their fields of application, when compared with their single-junction counterparts, given that the latter have their efficiency limited a priori.

### 3.1. Bandgap versus Efficiency

In order to grasp why single-junction cells are limited efficiency-wise, one has to fathom how the bandgap is of paramount importance when discussing solar cells.

Taking into consideration a single-junction solar cell with bandgap $W_{G}$, only photons with their energy higher or equal to $W_{G}$ are absorbed. Photons for which the energy is higher than the bandgap $W_{G}$, there is a certain amount of energy that is in excess and will be lost, an phenomenon also known as thermalization losses. This means that the energy that will be effectively converted into electric current will be just a portion of the photon’s total energy. With this, it is evident that the device will only operate at maximum efficiency when the photon’s energy, $W_{ph}$, is equal to the bandgap $W_{G}$. Alas, when considering the wide spectrum of sunlight, absorbing just the photons of a specific wavelength imposes quite a limitation on the overall efficiency of the cell.

In trying to solve this problem, a few solutions were developed. One of them is broadly used today in the PV industry: the concept of multi-junction solar cells. Instead of trying to make the cell operate only at a specific wavelength, one could try to divide the light spectrum into several spectral sections and associate a subcell with an appropriate bandgap to each one of them. This way, every subcell would have the unique function of absorbing photons of a specific wavelength range.

**Figure 2:**
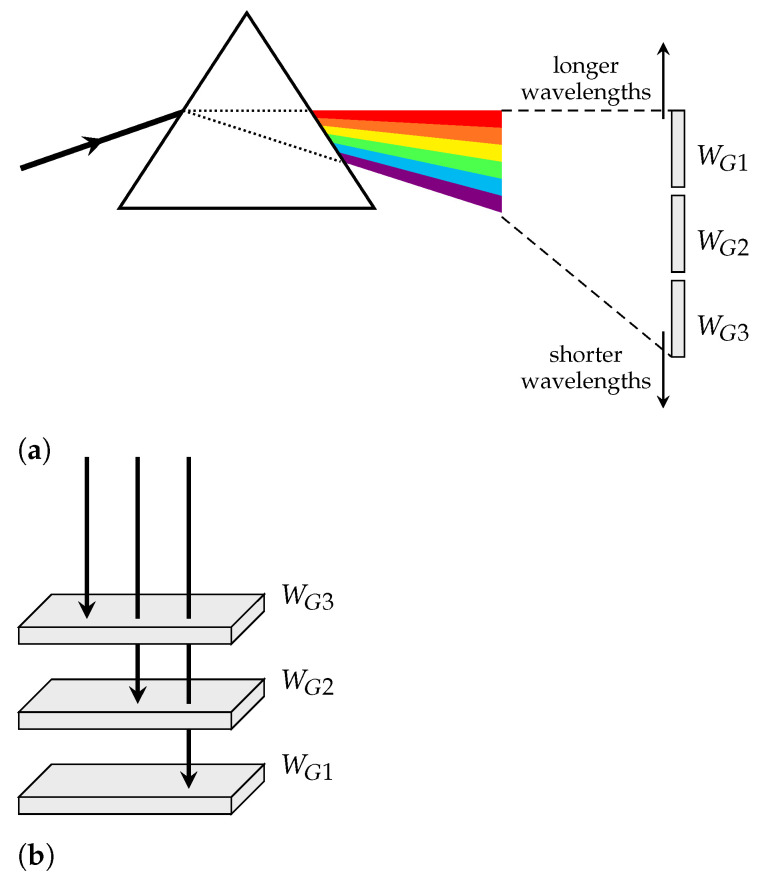
Spectral splitting approaches: (**a**) Spatial distribution, with the use of a prism, and (**b**) Stacked distribution of a 3-junction cell.

Now, there are different approaches to solve the problem and split the sunlight’s spectrum. The first is a quite intuitive one, called the spatial distribution method, illustrated on Figure 2a, and consists in using a prism to separate a beam of white light into several different wavelengths and spatially arranging subcells with different bandgap values accordingly.

Even though the spatial distribution is employed in some CPV (concentrator photovoltaic) systems, there are some difficulties associated when using this method. The approach that is broadly used nowadays when designing MJ solar cells is the stacked distribution, as presented in Figure 2b. This method consists in stacking the subcells on top of each other by order of bandgap, so the subcell with a lower bandgap is placed on the bottom of the cell and the one with a higher bandgap is placed on the top. This way, the high energy photons can be absorbed right on top of the cell by subcells with high bandgap values, forcing the low energy photons to penetrate further into the lower layers, where the low bandgap subcells are placed. As a result, the photons will be efficiently distributed and absorbed throughout the stack, increasing overall performance.

For this reason, the choice of bandgap combinations is a decisive step in multi-junction design. Given that III-V semiconductors show high versatility in possible bandgap combinations, they are one of the best choices for designing state-of-the-art solar cells. Bearing this in mind, Fraunhofer ISE developed the etaOpt software, capable of predicting cell efficiencies based on how many p–n junctions they are made of and what are their respective bandgaps. According to the results obtained from etaOpt, the efficiency can increase substantially with the number of p–n junctions, however, this gain is dampened for higher counts, i.e., a jump from 2 to 3 junctions provides a much larger increase than one from 5 to 6 junctions [29]. Knowing this data *a priori* is quite important for manufacturers, since the amount of efficiency gained may not justify higher production costs that derive from augmenting the number of p–n junctions.

### 3.2. Bandgap versus Lattice Constant

The choice of an appropriate bandgap does not take into account only the spectral regions, but also the choice of the lattice constant, since one depends on the other. This selection determines the structure of a MJ solar cell—if the materials all have, approximately, the same lattice constant, the cell is said to be lattice-matched; on the contrary, when the materials have different lattice constants, one says that the cell is lattice-mismatched or metamorphic (MM).

This distinction is significant when discussing solar cell design, given that stacked materials with different lattice constants may create dislocations, which can ruin the quality of the material and thus its performance. The production of metamorphic cells has to consider appropriate strategies, such as step-graded buffers that make the transition between two materials with different lattice-constants less abrupt.

## 4. The GaInP/GaInAs/Ge Solar Cell

Regarding this work, it seems only relevant to discuss approaches in which the GaInP/GaInAs/Ge solar cells are utilized. The two most relevant examples are the lattice-matched triple-junction and the upright metamorphic structures [4,5].

### 4.1. III-V Solar Cell Designs

**Figure 3:**
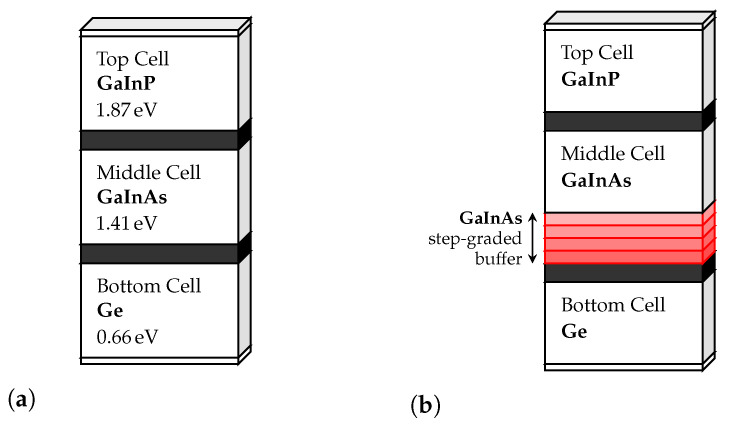
Simplified schematic of the triple-junction Ga${}_{0.5}$In${}_{0.5}$P/Ga${}_{0.99}$In${}_{0.01}$As/Ge cell: (**a**) lattice-matched, and (**b**) upright metamorphic approaches.

At the time this article is being written, the lattice-matched triple-junction Ga${}_{0.5}$In${}_{0.5}$P/ Ga${}_{0.99}$In${}_{0.01}$As/Ge, on Figure 3a, is the state-of-the-art cell for both space and terrestrial concentrator applications. The subcells are all lattice-matched to Ge, assuring that no dislocations are created. The cell itself consists of three main p–n junctions composed of GaInP, GaInAs, and Ge, stacked on top of each other, connected in series. The light falls on the GaInP subcell, which has the higher bandgap, as it was already explained previously. Each one of these subcells is connected through tunnel junctions with low resistance and high optical transmissivity coefficients. However, one of the problems of this approach is that the spectrum splitting is not optimal, resulting in an excessive current in the bottom Ge cell.

One possible way to counter this problem is to increase the absorption of photons in the upper cells, resulting in less current discrepancy. This can be achieved by lowering the bandgaps of the top and middle subcells by increasing the In composition in both ${\mathrm{Ga}}_{x}{\mathrm{In}}_{1-x}P$ and ${\mathrm{Ga}}_{x}{\mathrm{In}}_{1-x}\mathrm{As}$ materials. By doing this, the lattice constant also alters, and thus the materials no longer have the same lattice constant, making the cell lattice-mismatched or metamorphic (MM). This type of approach in monolithic structures may derive in dislocations that can harm material quality if no special measures are taken. In the case of the upright metamorphic TJ GaInP/GaInAs/Ge cell, presented in Figure 3b, one of those measures is to implement a GaInAs graded buffer between the middle and bottom cells, so that the lattice constant increases gradually and not abruptly.

### 4.2. Simulating the LM State-Of-The-Art Cell

In order to simulate this cell, one has to take into account that several companies are currently researching various approaches to its development, the two most important being Fraunhofer ISE and Spectrolab, Inc.

In this work, the approach that was utilized is identical to the one used at Spectrolab, where this cell already demonstrated an efficiency of 32% under 1-sun (AM1.5G spectrum) [30]. Moreover, it is assumed a $1\;cm^{2}$ active area. While there is published research of this cell concerning some of its specific structural information, there are not many details available about doping and thickness values and the material compositions of each layer, given that all of these specifics are treated as proprietary information of Spectrolab.

**Figure 4:**
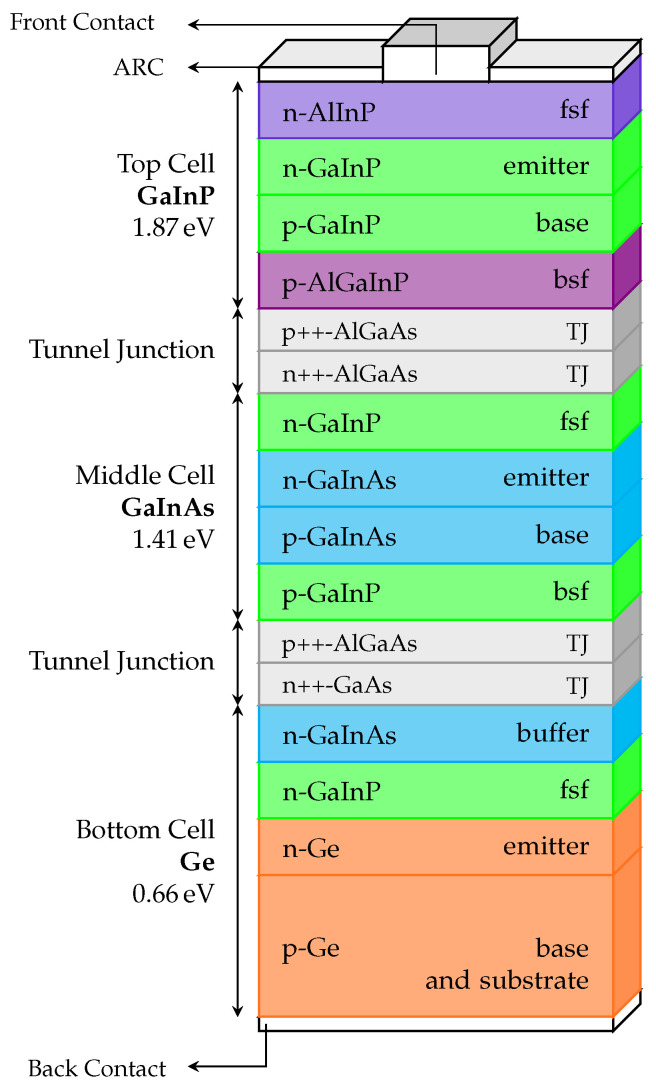
Detailed schematic of the simulated LM Ga${}_{0.5}$In${}_{0.5}$P/Ga${}_{0.99}$In${}_{0.01}$As/Ge solar cell.

Having as basis the detailed Ph.D. dissertation of Sharma [31], it was possible to put together an accurate model to simulate the cell. Some modifications were made to best adapt the cell to the one demonstrated by Spectrolab in the research paper of King et al. [30]. The simulated cell structure with all its layers is illustrated in Figure 4.

Firstly, to comprehend how the stacked cell works, it is necessary to perceive the role that each subcell plays in the monolithic cell by analyzing the materials that constitute each layer.

#### 4.2.1. The GaInP Top Subcell

Beginning from top to bottom, the first step was to simulate the GaInP top cell. This cell, as stated previously, has to absorb high energy photons, since it is on top of this cell that the light beams will fall onto. The Ga${}_{x}$In${}_{1-x}$P material is then chosen for its bandgap, which is $W_{G}=1.89\;\mathrm{eV}$ for a composition of x=0.5. This is a pretty high value that allows for the first high energy photons to be absorbed.

Besides the main p–n junction being composed of GaInP, the top subcell also contains two extra layers: the back-surface (BSF) and the window or front-surface (FSF) layers.

The window layer acts as an absorber layer, and thus it will have to have a high bandgap, small thickness, and a low series resistance. The material chosen can be the AlInP since it has a pretty high bandgap value and it is capable of being lattice-matched to the rest of the cell.

In contrast, the BSF layer exists to boost the short-circuit current of the cell, given that sharing the applied voltage across the n–p–p+ junctions minimizes the reflection of minority carriers and therefore leads to the decrease of the dark current. The material that is chosen for this is the quaternary AlGaInP.

#### 4.2.2. The GaInAs Middle Subcell

The second subcell to be simulated is the middle GaInAs cell, which is based in the more simple GaAs solar cell. It is lattice-matched to all the components that form the whole monolithic cell, with the main ternary compound, Ga${}_{x}$In${}_{1-x}$As, having the composition x=0.99 since its lattice constant corresponds to an exact-match to Ge’s.

The subcell also has window and back-surface layers that are composed of highly-doped GaInP (composition of x=0.5) given the high optical output of this material.

#### 4.2.3. The Ge Bottom Subcell

Finally, the bottom subcell is made of a Ge substrate, instead of the typically used GaAs. This has two major advantages; firstly, Ge is cheaper than GaAs, and secondly, since Ge has a very low bandgap ($W_{G}=0.66\;\mathrm{eV}$) the thickness of the subcell can be reduced from around 300μm for the GaAs substrate to 170μm for the Ge substrate.

Apart from a GaInP window layer similar to the middle cell one, the subcell also has a buffer layer made of highly-doped n-GaInAs (composition of x=0.99) in order to reduce the ohmic contact between the bottom cell and the tunnel junction.

#### 4.2.4. I–V Characteristic of the Stacked Cell

With the subcells already demonstrated, the next step was to try and assemble all of them in a monolithic cell.

Besides stacking the subcells on top of each other and separating them with appropriate tunnel junctions (AlGaAs–GaAs and AlGaAs–AlGaAs), it was also necessary to emulate the resistivity between subcell–tunnel diode and tunnel diode p–n junctions. This is made by establishing ohmic contacts with extremely high resistances that act as boundary conditions.

Two simulation models were tested: the first one, the cell was simulated in a Finite Element Tool without the metal grid (MG) on top, and the front contact had the same horizontal extension of the rest of the cell layers. This approach is a 1-D model since the structure only varies in one direction (vertical). The results were, then, artificially high since the contact effects were not being considered; the second method was employed so that the model would consider contact effects of the metal grid. Ergo, the cathode (top electrode) became smaller and a cap layer made of n+-GaAs was put below it with good ohmic contact formation in mind. Since there is this variation in the horizontal axis now, the model is a 2-D model.

**Figure 5:**
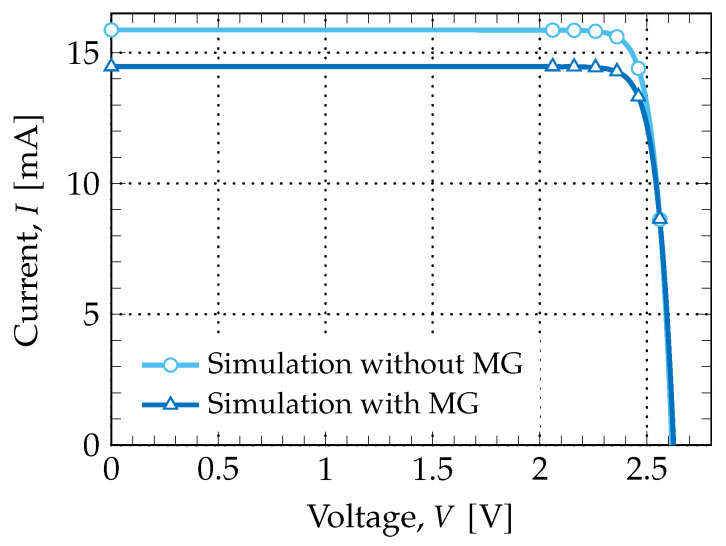
Simulated I−V characteristics of the stacked cell. All curves were obtained using the AM0 spectrum. Figures of merit are presented in Table 1.

Having as a reference the structure of the cell used at Spectrolab, the model developed in the Finite Element Tool was identical to the one depicted in Figure 4. In Figure 5, the obtained I−V characteristics are presented for both simulation models: 1-D model (without the MG) and 2-D model (with the MG). The most important figures of merit obtained from the simulated results are shown in Table 1; for comparison purposes, the experimental results from Spectrolab, Inc. [30] are also presented.

Analyzing the results, one can see that the best model to emulate the original cell’s behavior is the 2-D model, in which some of the device’s losses are being considered. The cell was emulated successfully to some extent: both the open-circuit voltage and short-circuit current were fairly replicated, which means that the overall structure (region materials, thickness, doping, etc.) was correctly modeled. Alas, both the fill-factor and efficiency were not consistent with the experimental results from Spectrolab. One explanation for this may be that losses were not properly accounted for in the final model, even taking the metal grid under consideration.

#### 4.2.5. External Quantum Efficiencies

The final test was to obtain the External Quantum Efficiency (EQE) from each subcell when stacked. This analysis provides a frequency response of the cell, which can be precious information to understand and further optimize solar cells.

Resorting to the optical bias method, it was possible to extract the individual EQE of each subcell. This method consists of saturating all the subcells simultaneously, except the one under test, so that the saturated junctions will not limit the current, while that the cell that is not saturated (the one under study) will determine the current value, and thus its EQE can be computed.

**Figure 6:**
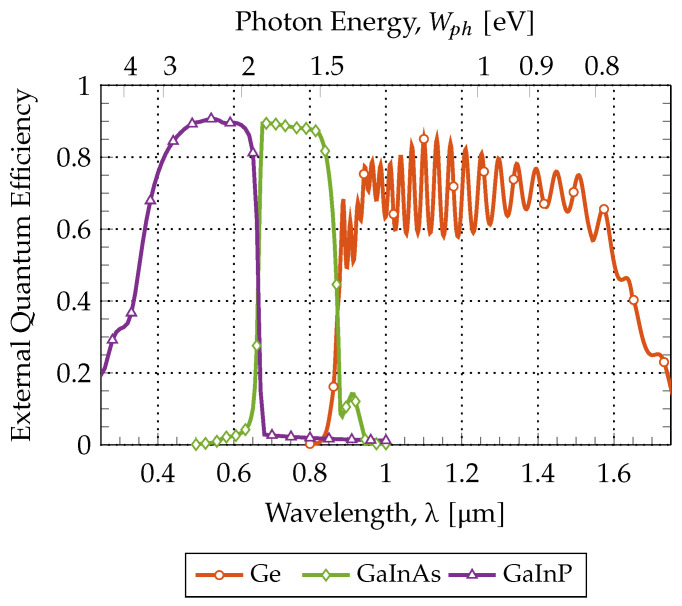
Simulated External Quantum Efficiencies of each subcell.

When computing the EQE, it is necessary to have in mind that each cell will only absorb in a very specific wavelength range, that strongly depends on the bandgap of the other subcells. This dependence is due to the fact that the light spectrum is being split by the stacked distribution. In the lattice-matched approach, the GaInP top cell absorbs photons with energy $W_{ph}>1.89\;\mathrm{eV}$, the GaInAs middle cell will absorb between the range of $1.89>W_{ph}>1.41\;\mathrm{eV}$ and, finally, the Ge bottom cell will absorb photons with energy $1.41>W_{ph}>0.661\;\mathrm{eV}$. All of this is well illustrated in Figure 6, in which the simulated results in the Finite Element Tool are presented.

### 4.3. Temperature Effects

Temperature, naturally, is one of the most important factors when studying the behavior of semiconductors. This way, solar cells are usually tested for a nominal operating cell temperature (NOCT) of 25°C, which is generally approximated to T=300K in absolute temperature values.

However, the photovoltaic cells under study have to be designed to withstand the extreme temperatures that only space can bestow. These temperatures can go from very high temperatures (HIHT (high intensity high temperature) missions) and deep-space temperatures like −170°C, which is the cell temperature for Saturn-orbit missions. Therefore, it makes sense to try and emulate the cell under these conditions.

#### High and Low Temperatures

To try and perceive how high temperatures affect solar cell performance, a simulation was run first for T=300K and then for higher temperatures, in intervals of 50K, to the final temperature of T=500K. Besides studying the cell’s behavior at high temperatures, it is also important to understand how they perform at temperatures below 0°C. Even if some parameters variances can be expected, namely the increase in the open-circuit voltage and overall efficiency, there is some interest in how they vary for low temperatures.

**Figure 7:**
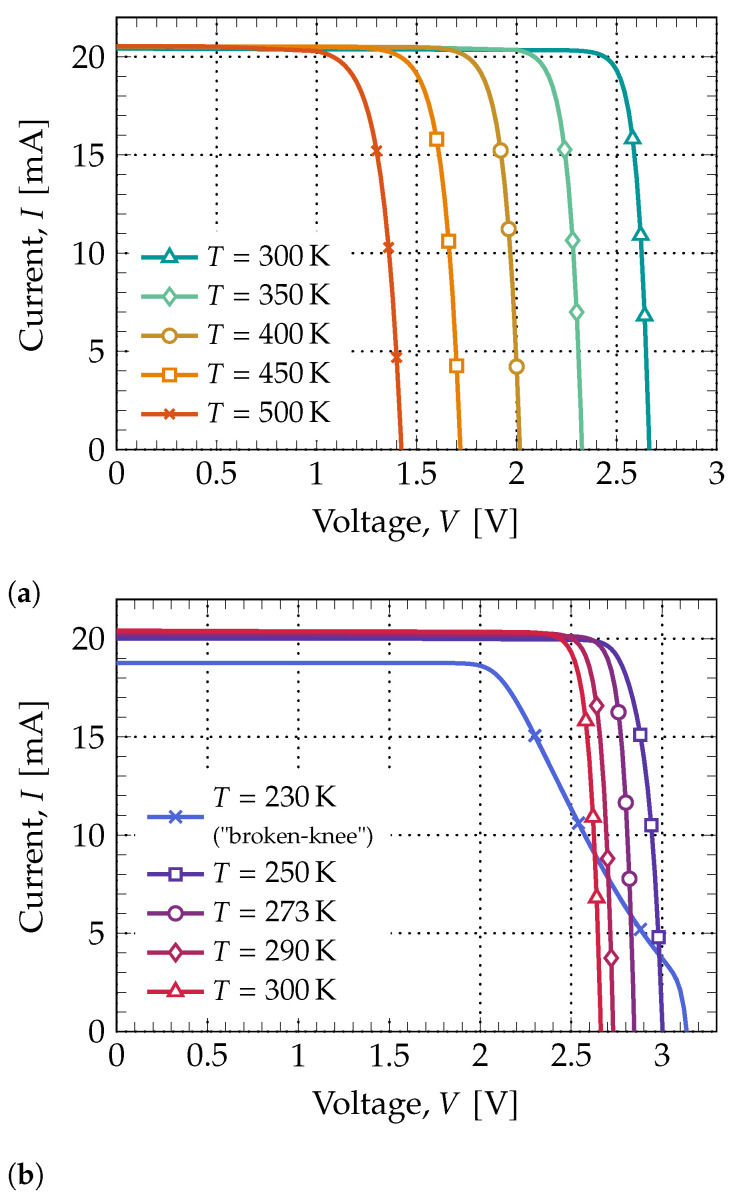
Tandem cell’s I−V characteristics obtained for two different intervals of temperature: (**a**) high temperature range, from T=300K to T=500K, and (**b**) low temperature range, from T=250K to T=300K. All curves were obtained using the AM0 spectrum.

The extracted I−V curves for both ranges of temperature are illustrated in Figure 7, with the most relevant figures-of-merit from both plots (Figure 7a,b) being registered in Table 2.

Both the open-circuit voltage and short-circuit current behave as expected: $I_{SC}$ has an insignificant variance whereas the $V_{OC}$ decreases substantially as temperature increases.

As for the efficiency and fill-factor, they both decrease as temperature rises, however, this is only valid to a certain point. As the array temperature gets colder, the variance in certain parameters begins to be non-linear. This is because, as temperature decreases, carriers start to enter the state of “freeze-out”, in which there is not enough thermal energy for the dopants to be fully ionized, and thus there will be a shortage of charge carriers. Another issue is the phenomenon called “broken-knee” or “double-slope”, in which the I−V characteristic becomes degraded, generating a great reduction in the fill-factor and efficiency—this can be seen in the obtained curve for T=230K.

Notwithstanding, colder environments, to a certain extent, are good for solar cells since there is a boost in the overall performance; the obtained results confirm the need for some PV panels to have cooling systems installed so that the power conversion efficiency is maximized.

## 5. Cell Optimization

With the lattice-matched GaInP/GaInAs/Ge solar cell properly reproduced and simulated, an overall optimization of the cell is attempted. In order to do this, two studies on how thickness and doping affect the overall performance of the cell were made. The first study takes into account the top and middle subcells and their respective thicknesses. The second study will take into account the doping of the GaInP top subcell. The properties of the whole cell were maintained constant with the default, previously simulated parameter values.

Considering that the cell that was being simulated up to this point was optimized for CPV (concentrator photovoltaic) applications, this work will attempt to perform an optimization for space applications in LEO (low-Earth orbit, <1000km) missions. The spectrum utilized was the AM0 and the cell temperature was T=300K.

### 5.1. Thickness Variation

When varying the cell thickness, it is necessary to select which layers are going to be altered. Since the photocurrent of the entire cell is determined by the top cell, the first layers to be chosen were the GaInP- base and emitter layers. The BSF and FSF layers were not altered, since their values were already at the minimum possible. The main goal of this study is to choose thickness values that establish a compromise between efficiency and size of the cell, given that the less cell bulkiness the better.

The first test consisted in varying both base and emitter thicknesses of the top cell and evaluate the efficiency, η, improvement. Other parameters like short-circuit current, $I_{SC}$, open-circuit voltage, $V_{OC}$, fill-factor, FF, and the variation in efficiency, $\Delta_{\mathrm{Eff}.}$, were also registered. Both default (gray) and best (green) obtained results for the first test are displayed in Table 3. The best efficiency achieved was 31.80% which in comparison to the initial value of 31.76% corresponds to an improvement of +0.1107%.

The second test was analogous to the first, except it was made considering only the middle subcell thickness. Once again, the BSF and FSF layers were not altered, varying only the thickness of both GaInAs- base and emitter layers. The top GaInP layers’ thicknesses were the initial ones, without employing the optimization of the first test. The default values along with the best-obtained results are presented in Table 4. Alas, in this case, the best-obtained results (in green) correspond to a thickness increase of 0.5μm in the base thickness. Since a bulkier cell is not the desired outcome, the second-best results (red) that achieved an efficiency of 31.8036% were chosen. This efficiency value corresponds to an improvement of +0.1098%.

**Figure 8:**
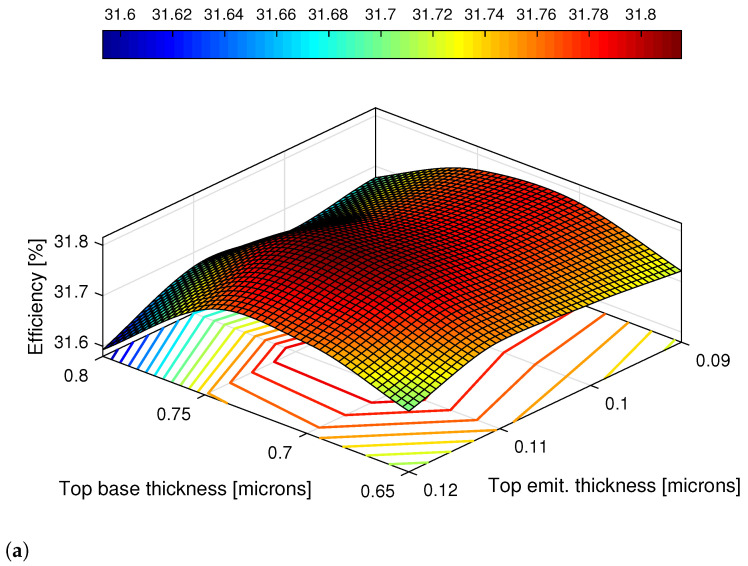

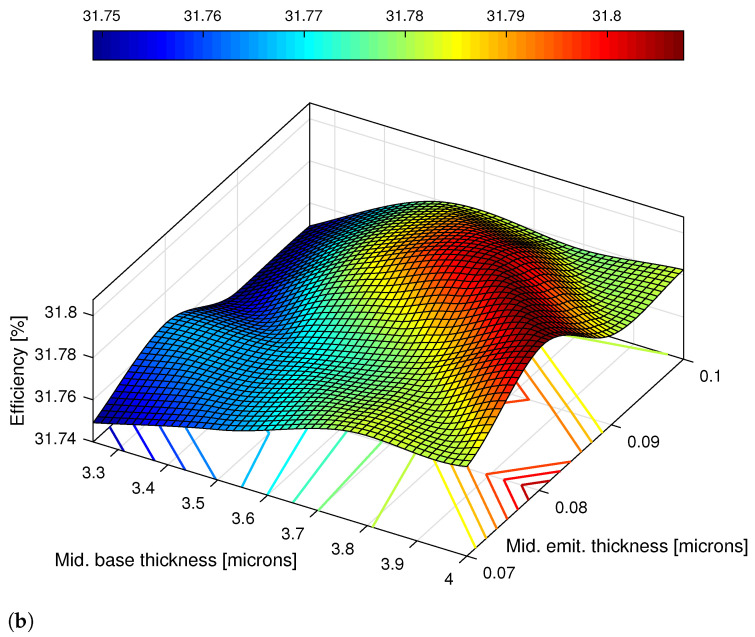
Graphic display of the obtained results for the: (**a**) first test: top GaInP subcell, and (**b**) middle GaInAs subcell. Both 3D plots were obtained using the 3D fitted surface with the cubic method of MATLAB Curve Fitting Toolbox.

All of the obtained results for both tests are illustrated in two 3D plots, in which one can observe how cell the layers’ thicknesses affect the overall performance of the cell. The 3D surface plots are presented in Figure 8 and were made resorting to the Curve Fitting Tool of MATLAB^©^.

With this visual aid, it fairly clear that for the first test (Figure 8a), the efficiency depends on both GaInP- base and emitter thicknesses, being apparent that higher efficiencies concentrate in a range of values that are roughly in the center of the plot.

Similarly, analyzing the 3D plot for the second test (Figure 8b) it is evident that the higher the middle subcell’s base thickness, the higher the efficiency. Unlike the first test, the GaInAs-base thickness is predominant in how the efficiency varies.

Finally, the best results from both tests were simulated, so that both subcell optimizations could be taken into account. The obtained parameters were: $I_{SC}=18.5943\;\mathrm{mA}$, $V_{OC}=2.6276\;V$, FF=88.98% and an efficiency of η=31.8431%, which translates in an improvement of 0.2343%, in comparison with the initial value.

### 5.2. Doping Variation

The second and final study was designed to evaluate how doping alters the performance of the cell. This last simulation is run with the best thickness values obtained in the first study.

Only the top cell’s base and emitter layers are going to be contemplated in this study. Once more, Table 5 shows the best-obtained results of doping variation for the GaInP- base and emitter layers. The best obtained efficiency was 33.0194%, which corresponds to a total improvement of +3.9368% of the very first efficiency value that was η=31.7687% (refer to Table 3 and Table 4).

The doping values that were simulated were carefully chosen, given that the higher the doping, the lower the potential barrier to be overcome, making higher efficiencies possible to achieve. However, this efficiency increase can not be indefinite, since the minority carrier lifetime and diffusion length decrease with doping increase [32]. Hence, searching for the optimal doping value that increases efficiency without degrading the electronic properties of the semiconductor is of paramount importance. Values past $2.00\times 10^{18}\;{\mathrm{cm}}^{-3}$ for the base and $1\times 10^{19}\;{\mathrm{cm}}^{-3}$ were not chosen, given that simulations run with doping values higher than these resulted in deterioration of the I−V curve.

**Figure 9:**
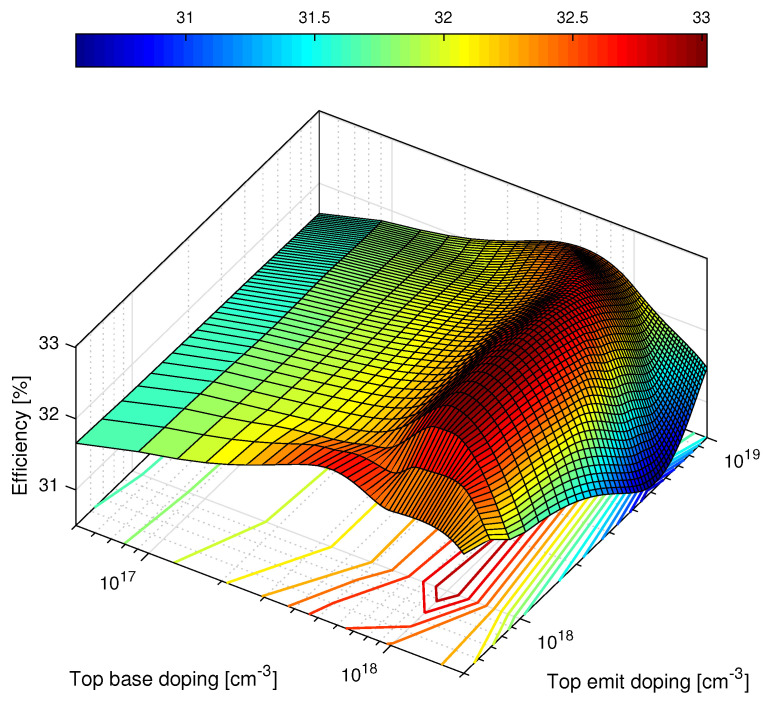
Graphic display of the obtained results of the second study. The 3D fitted surface with cubic method (MATLAB^©^).

Analogously to the first study, a 3D fitted cubic surface of the results was plotted and it is illustrated in Figure 9. It is clear that the base doping is predominant in efficiency variation; as it increases, efficiency values increase, reaching a peak region in which the efficiency is the highest possible. Beyond those values, there is an abrupt drop in the short-circuit current and open-circuit voltage, resulting in an efficiency reduction.

This concludes the optimization of the cell for operation under the AM0 spectrum, at the nominal temperature of T=300K. As it has already been mentioned, temperatures in space can oscillate from extremely low to very high temperatures (sometimes in the same mission), and so each PV array must be optimized in accordance with the conditions it is planned to operate at.

## 6. Conclusions

The main aim of this work was to create a model so that a triple-junction state-of-the-art solar cell could be emulated and then analyzed with accuracy, without the need to resort to more advanced, and expensive, simulation technologies.

Comparing the simulated results with the actual experimental results by Spectrolab, Inc. one could say that the main goal was achieved, and the cell was emulated successfully. Both the open-circuit voltage and short-circuit current were fairly replicated, which means that the region materials, thickness, and doping were correctly modeled.

However, both the fill-factor and efficiency were not consistent with their experimental counterparts; this may have to do with the fact that losses were not properly accounted in the modeled cell since the only loss mechanisms present were the metal grid and the back/front contacts, and the fact that complex refractive indices were used in simulation (the imaginary part accounts for losses). Experimental values are calculated by appropriate measuring devices, such as multimeters, connecting them in series/parallel to a resistor, which in turn is connected to both terminals of the cell. This results in part of the losses not being accounted for in the simulation.

Other relevant differences are the external quantum efficiencies that were obtained for each subcell, in contrast with the experimental curves from Spectrolab, Inc. [33]. This is due mainly to the use of refractive indexes that do not correspond to the exact composition of a certain material. For instance, the most obvious difference is between the simulated and experimental frequency responses in the middle cell; this discrepancy may reside in the fact that the only refractive index available (from the databases) is not a rigorous match for the composition of x=0.99 in Ga${}_{0}.99$In${}_{0.01}$As. This explanation is valid for other ternaries used as well.

Furthermore, bearing in mind that temperature plays a significant part in semiconductor performance, a test to evaluate how temperature influences the cell was also conducted. Most of the published research on how the GaInP/GaInAs/Ge solar cell behaves under different temperatures only has into consideration the higher range of temperature, given its paramount use in concentrator photovoltaic systems. With spacecraft implementation in sight, it was thought to be relevant to verify how the cell behaviors at low temperatures. Even though some plausible results were obtained, simulations in temperatures below 230 K did not obtain convergence, considering that the cell’s design was not prepared for such low-temperature environments.

Finally, an optimization of the GaInP/GaInAs/Ge LM cell was also conducted. In this optimization, certain cell parameters were tweaked so it could reach its maximum potential for a 1-AM0 incidence. This could prove of some value for the photovoltaic industry that is dedicated to the manufacturing of solar cells for space applications, given that the doping can significantly boost the cell’s efficiency.

Regarding the simulation times, depending on the mesh fineness and the voltage step that are being employed, the whole model takes roughly six minutes to simulate with Newton’s method. This may be an advantage over more complex and detailed ways of simulation that are more time-consuming if the main objective is simply to obtain the major figures-of-merit of the cell.

## Figures and Tables

**Table 1:** Comparison of experimental and simulation values for the stacked cell.

	Experimental	Simulation	
	**(Spectrolab)**	without MG *	with MG *
$I_{SC}\;\left[\mathrm{mA}\right]$	14.37	15.8771	14.4693
$V_{OC}\;\left[V\right]$	2.622	2.6296	2.6248
$V_{MP}\;\left[V\right]$	2.301	2.39	2.38
*FF*	0.85	0.89	0.88
Eff.[%]	32.0	36.9	33.65

* metal grid.

**Table 2:** Measured values for both temperature ranges: high and low temperatures, from the respective plots (a) and (b), displayed in Figure 7.

	Figure 7a					Figure 7b			
	230K	250K	273K	290K	300K	350K	400K	450K	500K
$I_{SC}\left[\mathrm{mA}\right]$	18.7692	19.9959	20.2036	20.3397	20.4106	20.5206	20.5199	20.5445	20.5342
$V_{OC}\left[V\right]$	3.1344	3.0021	2.8466	2.7307	2.6627	2.3263	2.0162	1.7189	1.4237
FF	0.64	0.88	0.91	0.90	0.89	0.87	0.85	0.81	0.77
Eff.[%]	27.77	38.79	38.25	36.72	35.73	30.59	25.74	21.05	16.46

**Table 3:** First study, first test: Top GaInP subcell thickness variation of the p-base and n-emitter layers.

Base [μm]	Emit. [μm]	$I_{\mathrm{SC}}$ [mA]	$V_{\mathrm{OC}}$ [V]	FF	η [%]	$\Delta_{\mathrm{Eff}.}$ [%]
0.75	0.10	18.6750	2.6251	0.8847	31.7687	0.0000
0.70	0.11	18.5956	2.6254	0.8893	31.8039	+0.1107

Default values; Best obtained values.

**Table 4:** First study, second test: Middle GaInAs subcell thickness variation of the p-base and n-emitter layers.

Base [μm]	Emit. [μm]	$I_{\mathrm{SC}}$ [mA]	$V_{\mathrm{OC}}$ [V]	FF	η [%]	$\Delta_{\mathrm{Eff}.}$ [%]
3.50	0.08	18.6750	2.6251	0.8847	31.7687	0.0000
4.00	0.08	18.6744	2.6272	0.8851	31.8074	+0.1220
3.75	0.09	18.6861	2.6262	0.8847	31.8036	+0.1098

Default values; Best obtained values; Second best values.

**Table 5:** Second study: doping variation of the p-base and n-emitter layers in the top subcell.

Base $\left[{\mathrm{cm}}^{-3}\right]$	Emit. $\left[{\mathrm{cm}}^{-3}\right]$	$I_{\mathrm{SC}}$ [mA]	$V_{\mathrm{OC}}$ [V]	FF	η [%]	$\Delta_{\mathrm{Eff}.}$ [%]
$1\times 10^{18}$	$1\times 10^{18}$	18.6371	2.6805	0.90	33.02	+3.9368
$1\times 10^{17}$	$5\times 10^{18}$	18.5943	2.6276	0.89	31.84	+0.2343

Default values; Best obtained values.

## Data Availability

Not applicable.
